# Evaluation of the ovicidal activity and fasciolicidal activity of the extract of ethyl acetate from *Artemisia ludoviciana* Nutt. spp. *mexicana* and of artemisinin against adult parasites of *Fasciola hepatica*

**DOI:** 10.1007/s00436-023-08052-6

**Published:** 2023-12-27

**Authors:** Alonso Ezeta-Miranda, José G. Avila-Acevedo, Yolanda Vera-Montenegro, Gerardo Francisco-Marquez

**Affiliations:** 1 Laboratorio de Fitoquímica, Unidad de Biología, Tecnología y Prototipos (UBIPRO), Facultad de Estudios Superiores Iztacala (FESI), Universidad Nacional Autónoma de México (UNAM), Av de los Barrios # 1, Tlalnepantla, Estado de México 54010 México; 2 Facultad de Medicina Veterinaria y Zootecnia (FMVZ), UNAM, Departamento de Pareasitología, Ciudad de México, 04510 México

**Keywords:** Adult flukes, Artemisinin, Extract, *Fasciola hepatica*, Ovicidal

## Abstract

The objective of this work was to evaluate the effect of the ethyl acetate extract from *A. ludoviciana* (EALM) and artemisinin against adult parasites and eggs of *F. hepatica*. For the ovicidal assay, cell culture plates with 24 wells were used, and 90 to 110 *F. hepatica* eggs were placed in each well. The eggs were exposed to concentrations of 100, 200, 300, 400, and 500 mg/L EALM and incubated for 16 days. Additionally, triclabendazole (TCBZ) was used as a reference drug at concentrations of 10 and 50 mg, and the response of artemisinin at concentrations of 10 and 20 mg was simultaneously assessed. Adult flukes were exposed to concentrations of 125, 250, 375, and 500 mg/L EALM. The results of the ovicidal action of EALM on the eggs showed that concentrations greater than 300 mg/L were significant, with ovicidal percentages greater than 60% observed on day 16 of incubation (*p* < 0.05). The maximum efficiency of EALM on adult flukes was reached 72 h post-exposure at a concentration of 125 mg/L (*p* < 0.05).

## Introduction

Fasciolosis is among the most serious liver diseases worldwide in the field of veterinary medicine and generates millions of dollars of economic losses (WHO 2020; FAO 2021). In addition, fasciolosis is a disease of serious public health concern, as it is an emerging zoonosis found in 70 countries; approximately 2.4 to 17 million people are infected worldwide and 1 billion more at risk (Sabourin et al. 2018; Fairweather et al. 2020). Over the years, fasciolosis has spread due to the growth of the global livestock industry and climatic factors favorable to bacterial adaptation and a greater geographical distribution of the intermediate host (Dargie 1987; Mas-Coma et al. 2005; Rojo et al. 2012; Rodríguez-Vivas et al. 2017; Sabourin et al. 2018; Alba et al. 2021; Chai and Jung 2022). Fasciolosis is caused by the fluke *Fasciola hepatica*. This parasite utilizes an indirect cycle and is located in the bile ducts of ruminants, swine, equines, rabbits, and humans (Javaregowda and Rani 2017). For decades, the main treatment for fasciolosis has involved chemotherapeutics (Olaechea et al. 2011). Unfortunately, due to the indiscriminate use of these drugs together with the poor prevention and diagnosis and genetic adaptation of the parasite, an increase in anthelmintic resistance to these drugs has occurred in different parts of the world (Moll et al. 2000; Olaechea et al. 2011; Kaplan and Vidyashankar 2012; Rojo et al. 2012; Ortiz et al. 2013; Hanna et al. 2015; Novobilsky et al. 2016; Ceballos et al. 2019; Kamaludeen et al. 2019; Romero et al. 2019; Fairweather et al. 2020; Kelley et al. 2020). An alternative substance to address this problem is natural products from plants with biological activity, which can be molecules or plant extracts. In Mexico, *Artemisia ludoviciana* Nutt. spp *mexicana* (estafiate), which belongs to the Asteraceae family, is recognized for its curative effect against diseases of the gastrointestinal tract (Andrade 2009; BDMTM/UNAM 2009). Extracts of different polarities obtained from the *A. ludoviciana* have demonstrated efficacy in vitro against young or recently excysted flukes, causing mortality greater than 90% at concentrations ranging from 125 to 500 mg/L (Álvarez et al. 2015; Ezeta et al. 2016). Recently, the effect of ethyl acetate extract from the *A. ludoviciana* on the tegument of recently excysted young flukes was demonstrated. Likewise, artemisinin was identified by HPLC/mass spectrometry as the major compound of the active extract (Ezeta et al. 2020). The objective of this work was to evaluate the effect of the ethyl acetate extract from *A. ludoviciana* and artemisinin against adult parasites and eggs of *F. hepatica*.

## Methodology

### Vegetal material

Healthy leaves of *A. ludoviciana* Nutt. spp. *mexicana* were collected in the vicinity of the Center for Teaching, Research and Extension in Tropical Livestock (CEIEGT), of the Faculty of Veterinary Medicine and Zootechnics (FMVZ), of the National Autonomous University of Mexico (UNAM), located in Martínez de la Torre—Tlapacoyan, municipality of Tlapacoyan, Veracruz, Mexico (19° 57′ 42″ N, 97° 12′ 39″ W). Taxonomic identification was performed in the herbarium of the Faculty of Higher Studies-Iztacala, UNAM (FESI-UNAM), and voucher number 2156 IZTA was assigned. The plant selection criteria were based on previous reports (Ibarra et al. 2012; Álvarez et al. 2015; Ezeta et al. 2020).

### Preparation of crude extract of *A. ludoviciana* Nutt. spp mexicana (EALM)

The leaves were dried at a constant temperature of 60 °C for 3 days and then ground. The ground material was macerated at room temperature with ethyl acetate for one week. The extract was filtered and concentrated to dryness under reduced pressure in a Heidolph^©^ Mod. Laborota 4000 rotary evaporator. Extracts were obtained once per week for 2 months and stored at 4 °C. The ethyl acetate extract obtained from *A. ludoviciana* (EALM) was used to perform the biological tests.

### Collection of adult specimens of *Fasciola hepatica* and in vitro tests

*F. hepatica* adults were obtained directly from infected bovine livers. The collection was performed in the municipal slaughterhouse of Toluca, State of Mexico. The specimens were first obtained and washed with phosphate-buffered saline to remove excess blood and bile and then placed in Roswell Park Memorial Institute (RPMI)-1640 medium at 37 °C to be transported to the Helminth Experimental Chemotherapy Laboratory of the Parasitology Department FMVZ-UNAM. Once in the laboratory, the flukes were washed several times with RPMI-1640 medium and placed in 20-ml tissue culture dishes in a medium created with 50% RPMI 1640 medium and 50% bovine serum. A mixture of antibiotics (100 IU penicillin + 100 mg/ml streptomycin) was added to the medium to prevent bacterial growth. Finally, 4 flukes were placed in each box (ratio of 1 fluke for every 5 ml of medium).

A stock solution was prepared with EALM at a concentration of 500 mg/L, which was previously dissolved in 100 µL of solvent and calibrated with distilled water to form the stock solution, from which dilutions were performed to obtain the corresponding concentrations. Adult flukes were exposed in triplicate to concentrations of 125, 250, 375, and 500 mg/L EALM, incorporating the corresponding controls for each solvent to confirm that the solvent did not affect the parasite; in addition, negative controls without any treatment were used. Additionally, triclabendazole (TCBZ) was used as a reference drug at concentrations of 10 and 50 mg (TCBZ was donated by the Dept. of Biopharmacy of the Faculty of Chemistry, UNAM), and the response of artemisinin (SIGMA- ALDRICH^©^, 98% purity, Ref. 361593–100 mg) at concentrations of 10 and 20 mg was simultaneously assessed. Once the different groups were exposed, the samples were incubated at 37 °C in a 5% CO_2_ atmosphere. (Burden and Hammet 1980; Hegazi et al. 2007; Helmy et al. 2008; Aguayo et al. 2018; Rehman et al. 2020; Sánchez et al. 2020; Guo et al. 2021). The test readings were obtained at 24, 48, and 72 h postexposure. To evaluate the effectiveness of the extract and the drugs on adult parasites, the mobility and mortality of each were considered according to the methodology and motility criteria described by Jeyathilakan et al. (2010). The control groups were observed, washed, and placed daily in a fresh culture medium to maintain their viability. Each experiment was performed in triplicate (De Mello et al. 2023).

All the procedures described were performed under aseptic conditions using a BG^©^ Mod. CFLV-130 laminar flow hood (Álvarez et al. 2015; WHO 2019; Ezeta et al. 2020, Rehman et al. 2020).

### Fasciolicide efficacy

The anti-fasciola efficacy was determined by comparing the survival of the treated group in relation to the control group as follows (Wood et al. 1995):

$$\mathrm{Efficacy}\left(\%\right)=\frac{\mathrm{Number}\;\mathrm{of}\;\mathrm{live}\;\mathrm{flukes}\;\mathrm{in}\;\mathrm{the}\;\mathrm{control}\;\mathrm{group}-\mathrm{Number}\;\mathrm{of}\;\mathrm{live}\;\mathrm{flukes}\;\mathrm{in}\;\mathrm{the}\;\mathrm{treated}\;\mathrm{group}}{\mathrm{Number}\;\mathrm{of}\;\mathrm{live}\;\mathrm{flukes}\;\mathrm{in}\;\mathrm{the}\;\mathrm{control}\;\mathrm{group}}\times100$$

### Ovicidal activity of EALM

To obtain *F. hepatica* eggs, gallbladders were collected from the livers of sheep affected by fascioliasis in the municipal slaughterhouse of Toluca, State of Mexico. The livers were transported to the laboratory at a temperature of 4 to 8 °C. Once in the laboratory, the bile contents were obtained aseptically and mixed with 400 to 500 ml of distilled water to settle the mixture and eggs for approximately 20 to 30 min. After that period, 2/3 of the volume of the mixture was decanted, and the samples underwent further gauging with distilled water. Then, the samples were left to settle for the same amount of time, which was performed until the liquid was as clear as possible and the eggs could be recovered from the bottom. The eggs were left at 4 °C for 24 h before ovicidal evaluation (Moazeni and Khademolhoseini 2016; Ceballos et al. 2019; Reigate et al. 2021). For the ovicidal assay, NUNC^©^ cell culture boxes with 24 wells were used, and 90 to 110 *F. hepatica* eggs were placed in each well. The eggs were exposed in triplicate to concentrations of 100, 200, 300, 400, and 500 mg/L EALM; control wells with solvent (ethyl acetate) and wells without treatment were used as control controls. Additionally, TCBZ was used at concentrations of 10 and 50 mg, and artemisinin at concentrations of 10 and 20 mg was assessed. Three replicates of each of the concentrations were carried out. Each of the boxes was covered with aluminum foil to protect them from light, and they were incubated for 14 and 16 days at a temperature of 28 °C and 80% humidity. After that period, they were exposed to 2 h of artificial light so that the miracidia would hatch. The ovicidal activity was evaluated according to formulas from the following (Najafi et al. 2017; Knepper et al. 2018; Machado et al. 2020):

$$\mathrm{Eggs\;hatched}\left(\%\right)=\frac{\mathrm{number}\;\mathrm{of}\;\mathrm{eggs}\;\mathrm{hatched}}{\mathrm{total}\;\mathrm{number}\;\mathrm{of}\;\mathrm{eggs}}\times100$$

$$\mathrm{Ovicidal}\;\mathrm{activity}\left(\%\right)=\frac{\%\;\mathrm e\mathrm g\mathrm g\mathrm s\;\mathrm h\mathrm a\mathrm t\mathrm c\mathrm h\mathrm e\mathrm d\;\mathrm i\mathrm n\;\mathrm c\mathrm o\mathrm n\mathrm t\mathrm r\mathrm o\mathrm l-\%\;\mathrm e\mathrm g\mathrm g\mathrm s\;\mathrm h\mathrm a\mathrm t\mathrm c\mathrm h\mathrm e\mathrm d\;\mathrm a\mathrm f\mathrm t\mathrm e\mathrm r\;\mathrm d\mathrm r\mathrm u\mathrm g\;\mathrm i\mathrm n\mathrm c\mathrm u\mathrm b\mathrm a\mathrm t\mathrm i\mathrm o\mathrm n}{\%\;\mathrm e\mathrm g\mathrm g\mathrm s\;\mathrm h\mathrm a\mathrm t\mathrm c\mathrm h\mathrm e\mathrm d\;\mathrm i\mathrm n\;\mathrm c\mathrm o\mathrm n\mathrm t\mathrm r\mathrm o\mathrm l}\times100$$

### Analysis of data

The data obtained were analyzed through the analysis of variance (ANOVA) test, Probit analysis, Dunnett’s test, and Kruskal‒Wallis test with a confidence interval of 95% to determine if there were statistically significant differences between the different treatments using SYSTAT v.12.0 32-bits (Systat Software, Inc. USA, 2008).

## Results

### EALM yields

Initially, 2.86 kg of green matter was collected, and when dried, 126 g of dry matter was obtained. At the end of the ethyl acetate extraction, a total of 17.85 g of EALM was obtained.

### Ovicidal activity

**Fig. 1:**
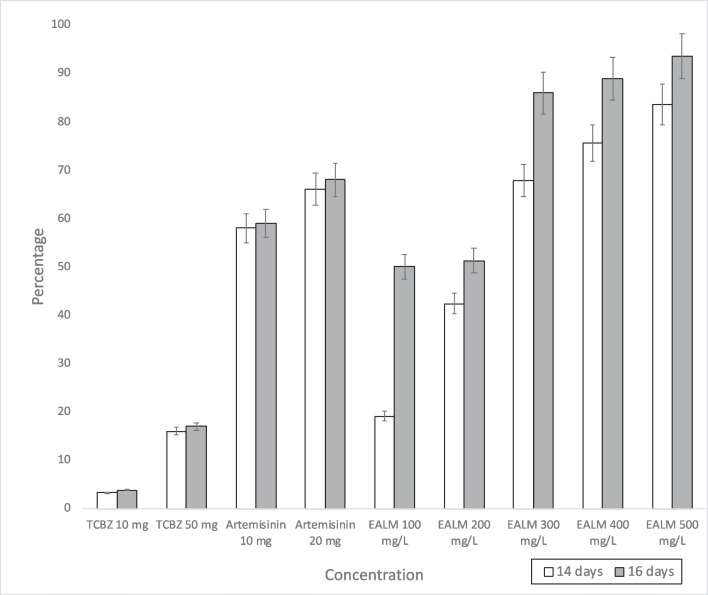
Percentage of ovicidal action of TCBZ, artemisinin, and EALM, by days of incubation, in the different study groups

The ovicidal activity was determined according to the number of hatched eggs in the different experimental groups. Regarding the percentages of ovicidal action, significant differences are observed between the reference compounds and EALM concentrations. Of the different concentrations of EALM, concentrations greater than 300 mg/L stand out, as ovicidal percentages greater than 60% were observed on both days of incubation (*p* < 0.05). The complete results of the percentage of ovicidal action of the eggs exposed to TCBZ, artemisinin, and EALM are shown in Table 1 and Fig. 1. The ovicidal effect increased according to the dose‒response relationship. The efficacy of EALM increased when the eggs were exposed for 2 more days, which represented a longer extract exposure time.

**Table 1:** Percentage of ovicidal action of TCBZ, artemisinin, and EALM, by days of incubation, in the different study groups

Days	Average % hatching in controls	Negative control	Solvent control (EtOAc)	Groups								
				TCBZ		Artemisinin		EALM (mg/L)				
				10 mg	50 mg	10 mg	20 mg	100	200	300	400	500
14	46.5	0	0	3.23	16.13	57.75	65.96	19.13	42.38	67.88	75.69	83.55
16	54.8	0	0	3.89	17.03	59.10	68.23	50.04	51.25	85.99	88.94	93.65

*EtOAc* ethyl acetate

**Fig. 2:**
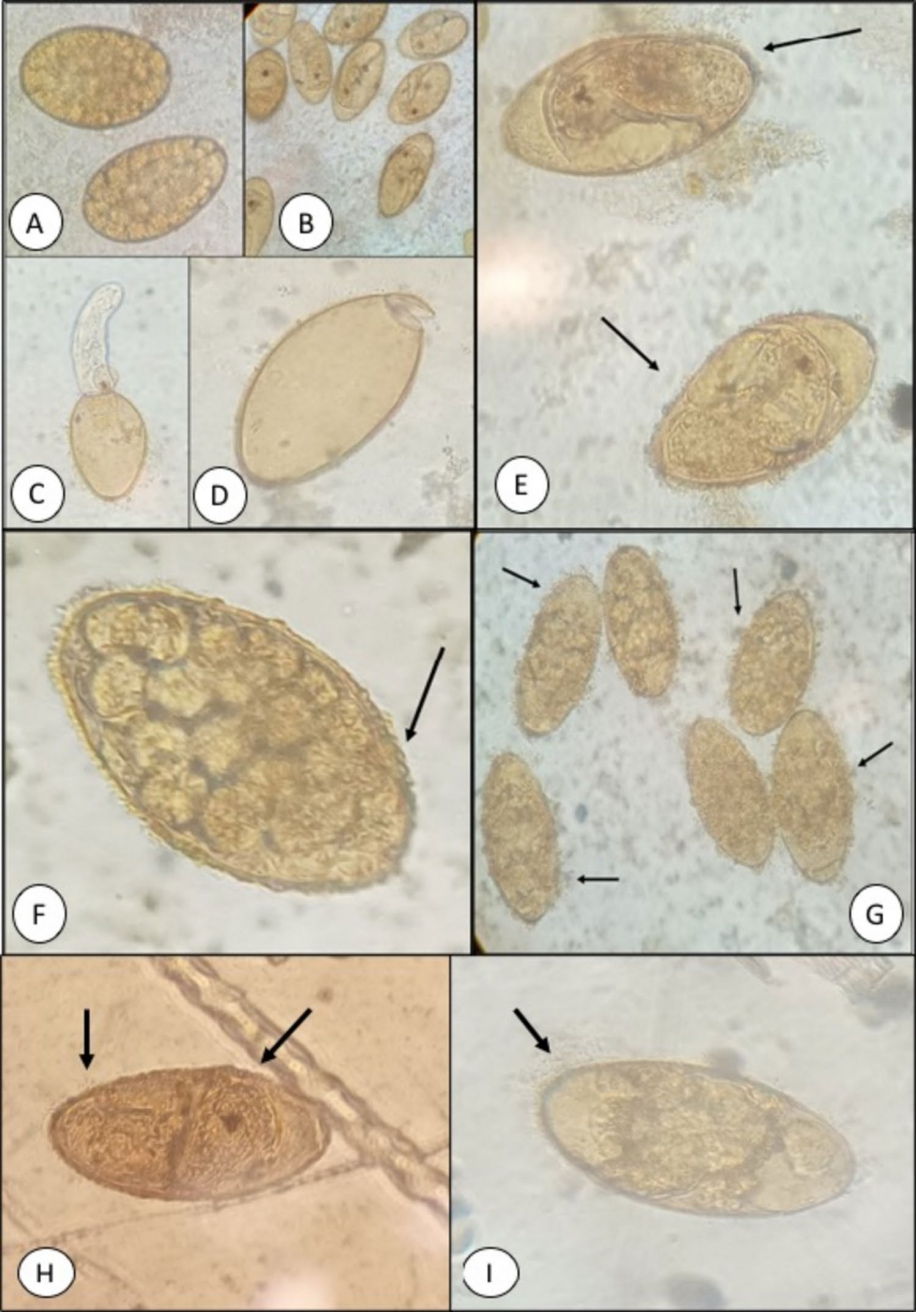
Observations obtained for the morphology of the eggs of *F. hepatica* exposed to EALM and artemisinin. **A** Eggs before being incubated; **B** Larval eggs after being incubated at 16 days; **C** miracidia emerging from the egg after being subjected to artificial light for 2 h; **D** empty egg after subjection to 2 h of artificial light; **E** egg of *F. hepatica* treated with 300 mg/L EALM; **F** egg of *F. hepatica* treated with 400 mg/L EALM; **G** egg of *F. hepatica* treated with 500 mg/L EALM; **H** egg of *F. hepatica* treated with 10 mg of artemisinin; and **I** egg of *F. hepatica* treated with 20 mg of artemisinin

The observations obtained for the morphology of the eggs exposed to EALM identified changes in relation to the control group are shown in Fig. 2. When the eggs were exposed to 300 mg/L EALM (Fig. 2E), miracidium formation was observed. However, changes in the internal and external morphology of the egg were detected. Presumably, these changes affected the development and hatching of the miracidia. Eggs exposed to EALM at a concentration of 400 mg/L (Fig. 2F) showed a change in the external egg morphology, especially in one of the egg poles (indicated with the arrow), and a change in the germ cells within the egg started to become noticeable. In an area in the group treated with 500 mg/L EALM (Fig. 2G), the outline of the eggs is not adequately distinguished (indicated with the arrows), and their content has begun to emerge, suggesting that their morphology was damaged. Regarding the eggs treated with artemisinin, eggs without larval development and others with miracidium formation were observed. However, in both cases, small changes could be seen in the egg periphery, suggesting that the compound caused damage and the egg integrity was affected (Fig. 2H and I).

### Fasciolicidal activity in adult flukes

**Fig. 3:**
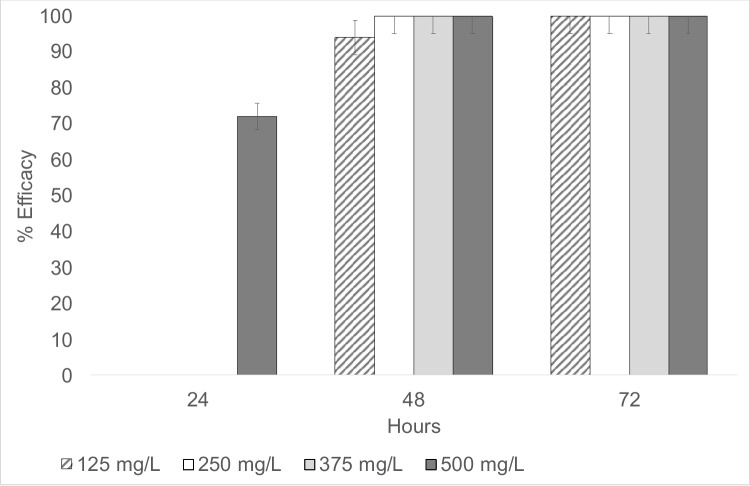
Evaluation of the different concentrations of EALM at different exposure times (*P* < 0.05)

The controls without any treatment did not suffer mortality during the duration of the experiment. In the groups treated with TCBZ and artemisinin, 100% efficacy was observed from the first 24 h at the two respective concentrations (*p* < 0.05). The fasciolicidal efficiencies of EALM at different concentrations showed differences, and 500 mg/L EALM was the only group that began to exhibit 72% efficacy at 24 h postexposure (*p* < 0.05). At 48 h postexposure, 125 mg/L showed 94% efficacy, and 250, 375, and 500 mg/L resulted in 100% efficacy (*p* < 0.05). Finally, at a concentration of 125 mg/L, the maximum efficacy was reached 72 h postexposure (*p* < 0.05) (Fig. 3). In general, an effect was observed in relation to concentration and exposure time. The complete fasciolicidal activities of EALM, TCBZ, and artemisinin against adult parasites are shown in Table 2. Regarding the different exposure times, an increasing efficacy began to be seen in the first 24 h of exposure, with concentrations of 500 mg/L reaching almost 100% efficacy 48 h postexposure (*p* < 0.05).

**Table 2:** Percentages of fasciolicidal efficacy in *F. hepatica* adults, triclabendazole, artemisinin, and EALM at different concentrations and exposure times

Hours	Concentration							
	TCBZ		Artemisinin		EALM			
	10 mg	20 mg	10 mg	20 mg	125 mg/L	250 mg/L	375 mg/L	500 mg/L
24	100	100	100	100	0	0	0	72 ± 0.092
48	100	100	100	100	94 ± 0.096	100	100	100
72	100	100	100	100	100	100	100	100

*TCBZ* triclabendazole

± denotes standard deviation

*P* < 0.05

### Lethal concentration (LCs) 50, 90, and 99 estimates from EALM

Probit analysis showed that the LC_50_, LC_90_, and LC_99_ values for the EALM with fasciolicidal efficacy in *F. hepatica* adults in vitro determined in this study are 80.1 mg/L, 310.9 mg/L, and 362.9 mg/L, respectively.

## Discussion

Previously, ovicidal assays of different parasites have been used to evaluate the efficacy of various compounds and plant extracts; recently, they have begun to be evaluated against *F. hepatica* eggs (Tunc et al. 2000; Kamaraj and Rahuman 2011; Moazeni and Khademolhoseini 2016; Moazeni et al. 2017; Najafi et al. 2017; Knepper et al. 2018; Machado et al. 2020). EALM had not been evaluated in this way, and this experiment showed an ovicidal effect on *F. hepatica* eggs. Presumably, there was damage to the eggshell, which may have affected its permeability. However, it is still necessary to determine in greater depth the type of damage that EALM has on the eggs to try to elucidate its mechanism of ovicidal action. In addition, certain drugs have been evaluated against *F. hepatica* eggs, such as ivermectin, artemisinin, and albendazole, all of which show ovicidal action, but the mechanism of action is still not clear (Moazeni et al. 2017). Regarding the evaluation of artemisinin and its ovicidal action, the observed damage was sufficient to alter egg integrity and prevent the development of miracidia and their hatching, although further studies are still necessary to visualize the nature of the damage to *F. hepatica*. In this experiment, although TCBZ was selected as the reference drug, it did not exhibit a considerable ovicidal percentage, in alignment with the results obtained by Álvarez et al. (2009). Additionally, the fasciolicidal effect of EALM shows its efficacy on adult parasites. Previously, its efficacy has been shown in newly excysted flukes, reaching fasciolicidal efficacy greater than 90% from the first 24 h postexposure (Ezeta et al. 2016, 2020). It is important to emphasize the differences that exist between young and adult flukes. From the time the definitive host is infected until the adult forms reach the liver, approximately 6 to 7 weeks pass. During this time, the changes in its tegument are influenced by the progression through the intestinal mucosa, peritoneum, and liver (Robinson et al. 2022; González et al. 2021). During their migration, young flukes, with the help of their glycocalyx, proteolytic systems, genetic expression, and changes in their energy metabolism, are able to better evade the host’s immune response, achieving greater adaptation and survival. All of this results in better development of the tegument, giving the flukes, among other things, better environmental protection (González et al. 2021). This may explain the differences between the fasciolicidal efficiencies of EALM in immature and adult forms of *F. hepatica*, especially at different exposure times where 100% efficacy is reached.

Additionally, the fasciolicidal effect of artemisinin has been demonstrated. Taking into account the previous work of Ezeta et al. (2020), the ethyl acetate extract of *A. ludoviciana* and its fractions point to the presence of artemisinin within them as the probable compound that generates the effect on parasites. Artemisinin is a constant metabolite within the genus *Artemisia* spp. and has been useful in treating *Plasmodium falciparum* infections in humans (Klayman 1985; Keizer and Utzinger 2007; Ferreira et al. 2011). Studies have been carried out with artesunate and artemether, which are semisynthetic derivatives of artemisinin, against *F. hepatica*, and an interruption in spermatogenesis in the adult flukes, affecting their reproductive system, was observed (O’Neill et al. 2009, 2015a, b, 2017). In another experiment, artemether caused extensive damage to the tegument of *F. hepatica* (Keiser and Morson 2008). However, none of these studies have directly evaluated artemisinin. Taking this into account, it is necessary to continue with studies that allow us to see the real damage to adult flukes caused by EALM and artemisinin, the compounds identified within the *A. ludoviciana* extract (Ezeta et al. 2020), to determine the affected area and compare the effect on the tegument of young and adult flukes. This would help in the ongoing effort to propose in vitro EALM as an integral option for the control of fasciolosis, covering different stages of parasite development and providing new insights into strategies against the fluke eggs.

## Conclusion

The extract of *A. ludoviciana* obtained from ethyl acetate has an ovicidal effect on *F. hepatica* eggs and fasciolicidal efficacy in adult forms of the parasite.

## Data Availability

All data supporting the findings of this study are available in the document.

## References

[CR1] Aguayo V, Valdes B, Espino AM (2018). Assessment of *Fasciola*
*hepatica* glutathione S-transferase as an antigen for serodiagnosis of human chronic fascioliasis. Acta Trop.

[CR2] Álvarez L, Moreno G, Moreno L, Ceballos L, Shaw L, Fairweather I, Lanusse C (2009). Comparative assessment of albendazole and triclabendazole ovicidal activity on *Fasciola*
*hepatica* eggs. Vet Parasitol.

[CR3] Álvarez JM, Ibarra F, Alonso MA, Vera Y, Ávila JG, García AM (2015). In vitro anthelmintic effect of fifteen tropical plant extracts on excysted flukes of *Fasciola*
*hepatica*. BMC Vet Res.

[CR4] Alba A, Vazquez AA, Hurtrez-Boussès S (2021). Towards the comprehension of fasciolosis (re)emergence: an integrative overview. Parasitol.

[CR5] Andrade CA (2009). Ethnobotanical study of the medicinal plants from Tlanchinol, Hidalgo, México. J Ethnopharmacol.

[CR6] Biblioteca Digital de la Medicina Tradicional (BDMTM). Universidad Nacional Autónoma de México (UNAM) (2009) Atlas de las Plantas de la Medicina Tradicional Mexicana (APMTM). Monografías, Estafiate. http://www.medicinatradicionalmexicana.unam.mx/apmtm/termino.php?l=3&t=artemisia-ludoviciana. Accessed 23 February 2023

[CR7] Burden DJ, Hammet NC (1980). *Fasciola*
*hepatica:* attempts to immunise rats using fluke eggs and in vitro culture products. Vet Parasitol.

[CR8] Ceballos L, Canton C, Pruzzo C, Sanabria R, Moreno L, Sanchis J, Suarez G, Ortiz P, Fairweather I, Lanusse C, Alvarez L, Martinez VM (2019). The egg hatch test: a useful tool for albendazole resistance diagnosis in *Fasciola*
*hepatica*. Vet Parasitol.

[CR9] Chai JY, Jung BK (2022). General overview of the current status of human foodborne trematodiasis. Parasitol.

[CR10] Dargie JD (1987). The impact on production and mechanisms of pathogenesis of trematode infections in cattle and sheep. Int J Parasitol.

[CR11] De Mello AB, Baccega BF, Martins FO, Da Rosa FNA, De Giacometi M, Da Fonseca RN, De Oliveira HS, Soares MP, Oliveira CB (2023). Microscopic alterations in *Fasciola*
*hepatica* treated with the essential oils of *Pelargonium*
*graveolens* and *Citrus aurantium*. Vet Parasitol.

[CR12] Ezeta MA, Vera MY, Ávila AJG, Álvarez MJM, Francisco MG (2016). In vitro fasciolicide activity of the raw extract of estafiate (*Artemisia*
*ludoviciana* Nutt. spp *mexicana*). Mex J Biotechnol.

[CR13] Ezeta MA, Vera MY, Ávila AJG, García BAM, Estrella PEA, Francisco MG, Ibarra VF (2020). Efficacy of purified fractions of *Artemisia*
*ludoviciana* Nutt. *mexicana* and ultrastructural damage to newly excysted juveniles of *Fasciola*
*hepatica* in vitro. Vet Parasitol.

[CR14] Fairweather I, Brennan GP, Hanna REB, Robinson MW, Skuce PJ (2020). Drug resistance in liver flukes. Int J Parasitol Drugs Drug Resist.

[CR15] Ferreira JFS, Peaden P, Keiser J (2011). In vitro trematocidal effects of crude alcoholic extracts of *Artemisia*
*annua*, *A.*
*absinthium*, *Asimina*
*triloba*, and *Fumaria*
*officinalis*. Trematocidal Plant Alcoholic Extracts Parasitol Res.

[CR16] Food and Agriculture Organization of the United Nations (FAO) (2021) Fasciolasis (liver fluke). Foodborne parasitic infections. https://www.fao.org/documents/card/en/c/cb1127en. Accessed 27 February 2023

[CR17] González MJ, Becerro RD, Siles LM (2021). Insights into *Fasciola*
*hepatica* juveniles: crossing the fasciolosis rubicon. Trends Parasitol.

[CR18] Guo A, Wang L, Meng X, Zhang S, Sheng S, Luo X, Huang W, Wang S, Cai X (2021). Extracellular vesicles from *Fasciola*
*gigantica* induce cellular response to stress of host cells. Exp Parasitol.

[CR19] Hanna REB, Mc Mahon C, Ellison S, Edgar HW, Kajugu PE, Gordon A, Irwin D, Barley JP, Malone FE, Brennan GP, Fairweather I (2015). *Fasciola*
*hepatica*: a comparative survey of adult fluke resistance to triclabendazole, nitroxynil and closantel on selected upland and lowland sheep farms in Northern Ireland using faecal egg counting, coproantigen ELISA testing and fluke histology. Vet Parasitol.

[CR20] Hegazi AG, Abd El Hady FK, Shalaby HA (2007). An in vitro effect of propolis on adult worms of *Fasciola*
*gigantica*. Vet Parasitol.

[CR21] Helmy MMF, Fahmy ZH, Sabry HY (2008). *Fasciola*
*gigantica*: evaluation of the effect of phenyl vinyl sulfone *in*
*vitro*. Exp Parasitol.

[CR22] Ibarra MS, Ibarra VF, Ávila AJG (2012) In vitro evaluation of fasciolicide activity with hexane, methanol and ethyl acetate with extracts processed and obtained from some mexican plants used in traditional medicine based on ethno botanical studies. Am J Plant Sci 3:506–511

[CR23] Javaregowda AK, Rani KB (2017). Chronic bovine fasciolosis associated cholangiolithiasis: a case report. J Parasit Dis.

[CR24] Jeyathilakan N, Murali K, Anandaraj A, Latha AR, AbdulBasith S (2010). Anthelmintic activity of essential oils of *Cymbopogan*
*nardus* and *Azadirachta*
*indica* on *Fasciola*
*gigantica*. Vet Anim Sci.

[CR25] Kamaludeen J, Graham BJ, Stephens N, Miller J, Howell A, Beesley N, Hodgkinson J, Learmount J, Williams D (2019). Lack of efficacy of triclabendazole against *Fasciola*
*hepatica* is present on sheep farms in three regions of England, and Wales. Vet Res.

[CR26] Kamaraj C, Rahuman AA (2011). Efficacy of anthelmintic properties of medicinal plant extracts against *Haemonchus*
*contortus*. Res Vet Sci.

[CR27] Kaplan RM, Vidyashankar AN (2012). An inconvenient truth: global worming and anthelmintic resistance. Vet Parasitol.

[CR28] Keiser J, Utzinger J (2007). Food-borne trematodiasis: current chemotherapy and advances with artemisinins and synthetic trioxolanes. Trends Parasitol.

[CR29] Keiser J, Morson G (2008). Fasciola hepatica: tegumental alterations in adult flukes following in vitro and in vivo administration of artesunate and artemether. Exp Parasitol.

[CR30] Kelley JM, Rathinasamy V, Elliot TP, Rawlin G, Beddoe T, Stevenson MA, Spithill TW (2020). Determination of the prevalence and intensity of *Fasciola*
*hepatica* infection in dairy cattle from six irrigation regions of Victoria, South-eastern Australia, further identifying significant triclabendazole resistance on three properties. Vet Parasitol.

[CR31] Klayman DL (1985). Qinghaosu (artemisinin): an antimalarial drug from China. Science.

[CR32] Knepper ZF, Machado PMA, Massia PK, Aires BME, Cunico W, Campos JJC, Pires GD, Silva NP, Oliveira HS, Machado SG (2018). Ovicidal *in*
*vitro* activity of 2-aryl-3-(2-morpholinoethyl)thiazolidin-4-ones and 2-aryl-3-(3-morpholinopropyl)thiazolidin-4-ones against *Fasciola*
*hepatica* (Linnaeus, 1758). Exp Parasitol.

[CR33] Machado PMA, Knepper ZF, Massia PK, Silveira PB, Antonio FR, Berne PN, Helena MR, Villareal VJP, Oliveira HS, Aires BME, Silva NP (2020). Ovicidal in vitro activity of the fixed oil of *Helianthus*
*annus* L. and the essential oil of *Cuminum*
*cyminum* L. against *Fasciola*
*hepatica* (Linnaeus, 1758). Exp Parasitol.

[CR34] Mas-Coma S, Bargues MD, Valero MA (2005). Fasciolasis and other plant-borne trematode zoonoses. Int J Parasitol.

[CR35] Moazeni M, Khademolhoseini AA (2016). Ovicidal effect of the methanolic extract of ginger (*Zingiber*
*officinale*) on *Fasciola*
*hepatica* eggs: an in vitro study. J Parasit Dis.

[CR36] Moazeni M, Saadaty AZS, Saharkhiz MJ, Jalaei J, Khademolhoseini AA, Esfand ASS, Alavi AM (2017). In vitro ovicidal activity of *Peganun*
*harmala* seeds extracton the eggs of *Fasciola*
*hepatica*. J Parasit Dis.

[CR37] Moll L, Gaasenbeek CPH, Vellema P, Borgsteede FHM (2000). Resistance of *Fasciola*
*hepatica* against triclabendazole in cattle and sheep in The Netherlands. Vet Parasitol.

[CR38] Najafi F, Rezaie S, Kia EB, Mahmoudi M, Khodavaisy S, Mohebali M, Gharagozlou MJ, Rokni MB, Mowlavi G (2017). In vitro assay of *Paecilomyces*
*lilacinus* biocontrol effects on *Fasciola*
*hepatica* eggs illustrated in scanning electron micrographs. Iran J Parasitol.

[CR39] Novobilsky A, Solis NA, Skarin M, Höglund J (2016). Assessment of flukicide efficacy against *Fasciola*
*hepatica* in sheep in Sweden in the absence of a standardised test. Int J Parasitol Drugs Drug Resist.

[CR40] Olaechea F, Lovera V, Larroza M, Raffo F, Cabrera R (2011). Resistance of *Fasciola*
*hepatica* against triclabendazole in cattle in Patagonia (Argentina). Vet Parasitol.

[CR41] O’Neill JF, Johnston RC, Halferty L, Brennan GP, Keiser J, Fairweather I (2009). Adult triclabendazole-resistant *Fasciola*
*hepatica:* morphological changes in the tegument and gut following in vivo treatment with artemether in the rat model. J Helminthol.

[CR42] O’Neill JF, Johnston RC, Halferty L, Brennan GP, Fairweather I (2015). Ultrastructural changes in the tegument and gut of adult *Fasciola*
*hepatica* following in vivo treatment with artesunate. Exp Parasitol.

[CR43] O’Neill JF, Johnston RC, Halferty L, Hanna REB, Brennan GP, Fairweather I (2015). A comparative study on the impact of two artemisinin derivatives, artemether and artesunate, on the female reproductive system of *Fasciola*
*hepatica*. Vet Parasitol.

[CR44] O’Neill JF, Johnston RC, Halferty L, Hanna REB, Brennan GP, Fairweather I (2017). Disruption of spermatogenesis in the liver fluke, *Fasciola*
*hepatica* by two artemisinin derivates, artemether and artesunate. J Helminthol.

[CR45] Ortiz P, Scarcella S, Cerna C, Rosales C, Cabrera M, Guzmán M, Lamenza P, Solana H (2013). Resistance of *Fasciola*
*hepatica* against triclabendazole in cattle in Cajamarca (Perú): a clinical trial and an *in*
*vivo* efficacy test in sheep. Vet Parasitol.

[CR46] Rehman A, Ullah R, Gupta D, Khan MAH, Rehman L, Beg MA, Khan AU, Abidi SMA (2020). Generation of oxidative stress and induction of apoptotic like events in curcumin and thymoquine treated adult *Fasciola*
*gigantica* worms. Exp Parasitol.

[CR47] Reigate C, Williams HW, Denwood MJ, Morphew RM, Thomas ER, Brophy PM (2021). Evaluation of two *Fasciola*
*hepatica* faecal egg counting protocols in sheep and cattle. Vet Parasitol.

[CR48] Robinson MW, Hanna REB, Fairweather I, Dalton JP (2022). Development of *Fasciola**hepatica* in the mammalian host. Fasciolosis.

[CR49] Rodríguez-Vivas RI, Grisi L, Pérez de León AA, Silva VH, Torres-Acosta JF, Fragoso SH, Romero SD, Rosario CR, Saldierna F, García CD (2017). Potential economic impact assessment for cattle parasites in Mexico. Review. Rev Mex Cienc Pecu.

[CR50] Rojo VFA, Meana A, Valcárcel F, Martínez VM (2012). Update on trematode infections in sheep. Vet Parasitol.

[CR51] Romero J, Villaguala C, Quiroz F, Laudaeta-Aqueveque C, Alfaro G, Pérez R (2019). Flukicide efficacy against *Fasciola*
*hepatica* of triclabendazole and nitroxynil in cattle of the central valley of Chile. Brazil J Vet Parasitol.

[CR52] Sánchez LCM, Trelis M, Jara L, Cantalapiedra F, Marcilla A, Bernal D (2020). Diversity of extracellular vesicles from different development stages of *Fasciola*
*hepatica*. Int J Parasitol.

[CR53] Sabourin E, Alda P, Vázquez A, Hurtrez-Bousses S, Vittecoq M (2018). Impact of human activities on fasciolosis transmission. Trends Parasitol.

[CR54] Tunc IB, Berger BM, Erler F, Dagli F (2000). Ovicidal activity of essential oils from five plants against two stored-product insects. J Stored Prod Res.

[CR55] Wood IB, Amaral NK, Bairden K, Duncan JL, Kassai T, Malone JB Jr, Pankavic JA, Reinecke RK, Slocombe O, Taylor SM, Vercruysse J (1995) World Association for the Advancement of Veterinary Parasitology (W.A.A.V.P.) second edition of guidelines for evaluating the efficacy of anthelmintics in ruminants (bovine, ovine, caprine). Vet Parasitol 58:181–213. 10.1016/0304-4017(95)00806-2 10.1016/0304-4017(95)00806-2 7571325

[CR56] World Health Organization (WHO) (2019) Technical document of the use of non-pharmaceutical forms of *Artemisia.* WHO Headquarters, Geneva, Switzerland, October, pp 1–21. Access https://www.who.int/publications/i/item/WHO-CDS-GMP-2019.14. Accesed 3 Mar 2023

[CR57] World Health Organization (WHO) (2020) Ending the neglect to attain the sustainable development goals: a road map for neglected tropical diseases 2021–2030. Geneva: World Health Organization, pp 106–109. Access https://www.who.int/publications/i/item/9789240010352. Accesed 5 Mar 2023

